# Avian pathogenic *Escherichia coli* infection causes infiltration of heterophilic granulocytes of chick tracheal by the complement and coagulation cascades pathway

**DOI:** 10.1186/s12917-023-03838-3

**Published:** 2023-12-08

**Authors:** Ziqi Li, Zhao Qi, Xiaoru Wang, Liting Lu, Haiyang Wang, Zhenjie He, Zhe Chen, Ying Shao, Jian Tu, Xiangjun Song

**Affiliations:** 1 Anhui Province Key Laboratory of Veterinary Pathobiology and Disease Control, College of Animal Science and Technology, Anhui Agricultural University, Hefei, 230036 PR China; 2 Anhui Province Engineering Laboratory for Animal Food Quality and Bio-safety, College of Animal Science and Technology, Anhui Agricultural University, Hefei, 230036 PR China; 3 Key Laboratory for Agri-Food Safety, School of Resource & Environment, Anhui Agricultural University, Hefei, Anhui 230036 PR China

**Keywords:** Avian pathogenic *Escherichia coli*, AE17, TMT-sequencing, Complement and coagulation cascades pathways, Pathogenicity

## Abstract

**Background:**

Avian pathogenic *Escherichia coli* (**APEC**) causes tracheal damage and heterophilic granulocytic infiltration and inflammation in infected chicks. In this study, we infected chick tracheal tissue with strain AE17 and produced pathological sections with proteomic sequencing. We compared the results of pathological sections from the APEC-infected group with those from the PBS control group; the pathological sections from the experimental group showed hemorrhage, fibrinization, and infiltration of heterophilic granulocytes in the tracheal tissue. In order to explore the effect on proteomics on inflammation and to further search for the caus.

**Results:**

The tandem mass tag-based (**TMT**) sequencing analysis showed 224 upregulated and 140 downregulated proteins after infection with the AE17 strain. Based on the results of KEGG in Complement and coagulation cascades, differential protein expression in the Protein export pathway was upregulated.

**Conclusions:**

With these results, we found that chemokines produced by the Complement and coagulation cascades pathway may cause infiltration of heterophilic granulocytes involved in inflammation, as well as antimicrobial factors produced by the complement system to fight the infection together.These results suggest that APEC causes the infiltration of heterophilic granulocytes through the involvement of the complement system with serine protease inhibitors.

## Background

Avian colibacillosis caused by Avian Pathogenic *Escherichia coli* (**APEC**) is widespread worldwide. It is mainly transmitted through the respiratory tract and often leads to collective illness in birds, causing local or systemic infection with high morbidity and mortality rates on large-scale farms and economic losses [1]. The colonization of respiratory airway epithelial cells is thought to be an important step in the pathogenesis of APEC, and colonization by APEC in avian respiratory epithelial cells has been demonstrated in several in vivo and in vitro assays [2]. After breaking the air-blood barrier, APEC enters the bloodstream and causes systemic infection; the initial step in APEC pathogenesis is adherence to the trachea and infection of chickens through the respiratory tract [3]. The results of previous experiments in our laboratory have confirmed that APEC infection is followed by pathological damage to the trachea [4].

Coexistence and interaction of hemostatic and inflammatory mediators after infection by APEC usually ensures successful host immune defense in a compromised barrier environment [5]. Among these, the complement system mediates a wide range of important biological functions, and there are significant similarities among many proteins that play enzymatic and regulatory roles in alternative and classical pathways [6]. There are three pathways of complement activation: the classical, the lectin, and the alternative pathway, all of which generate key enzymatic activities that in turn produce effector molecules of complement [7]. These complements generate an adaptive immune response, involving many immune cells [7]. These complements generate an adaptive immune response, involving many immune cells [8]. Heterophils are chicken leukocytes that protect the body from invading pathogens by phagocytosing and killing them. The results of the pathological sections in this experiment showed an infiltration of heterophilic granulocytes [9]. Avian heterophilic granulocytes are the major granulocytes in the circulating blood of most birds and are similar to mammalian neutrophils in that they are highly phagocytic. As the main participant and regulator of body immunity, heterophilic granulocytes play a crucial role in the chicken immune system. When the organism is invaded by pathogenic microorganisms, heterophilic granulocytes are recruited in large numbers to the damaged and inflammatory sites for phagocytosis and the killing of pathogenic microorganisms, acting as the first line of immune defense of the organism [10].

However, less research has been done on heterophilic granulocytes in chickens, and the mechanism underling the infiltration of heterophilic granulocytes, the main defense position of the body’s immunity, is still largely unclear. This makes it important to explore the polyinfiltration of heterophilic granulocytes. In this study, to investigate the infiltration of heterophilic granulocytes caused by APEC infection of chicken trachea, the pathological findings of chicken trachea after APEC infection and TMT sequencing were analyzed.

## Results

### Histopathological sections showed infiltration of heterophilic granulocytes

**Fig. 1:**
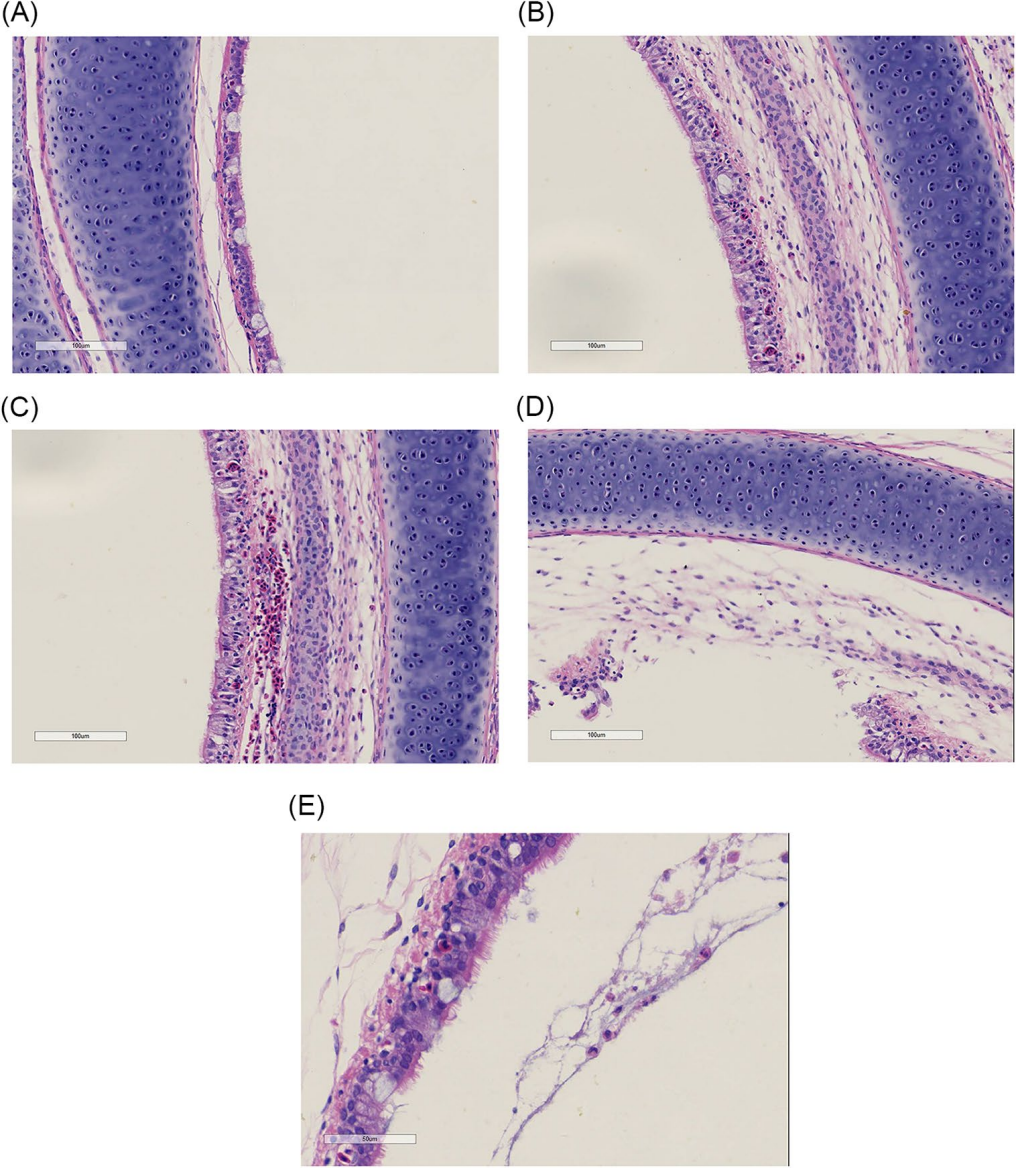
Pathological section findings following *Escherichia coli* infection. (**A**) Control group. (**B**) Infiltration of heterophilic granulocytes after infection. (**C**) Bleeding after infection. (**D**) Cell shedding after infection. (**E**) Heterophilic granulocytes with fibrin that appear after infection

To investigate the effect of AE17 on tracheal injury, the PBS group was used as a control group (Fig. 1A). The pathological sections in the experimental group showed symptoms of hemorrhage, with cell detachment and fibrinization (Fig. 1 C and 1D), suggesting that AE17 causes damage to the trachea, with a strong link to the serine protease inhibitors below. Figure 1B and E show the infiltration of heterophilic granulocytes, suggesting that AE17 causes infiltration of heterophilic granulocytes in the epithelial tissue of the trachea.

### Identification of differentially expressed proteins between AE17 and the control

**Fig. 2:**
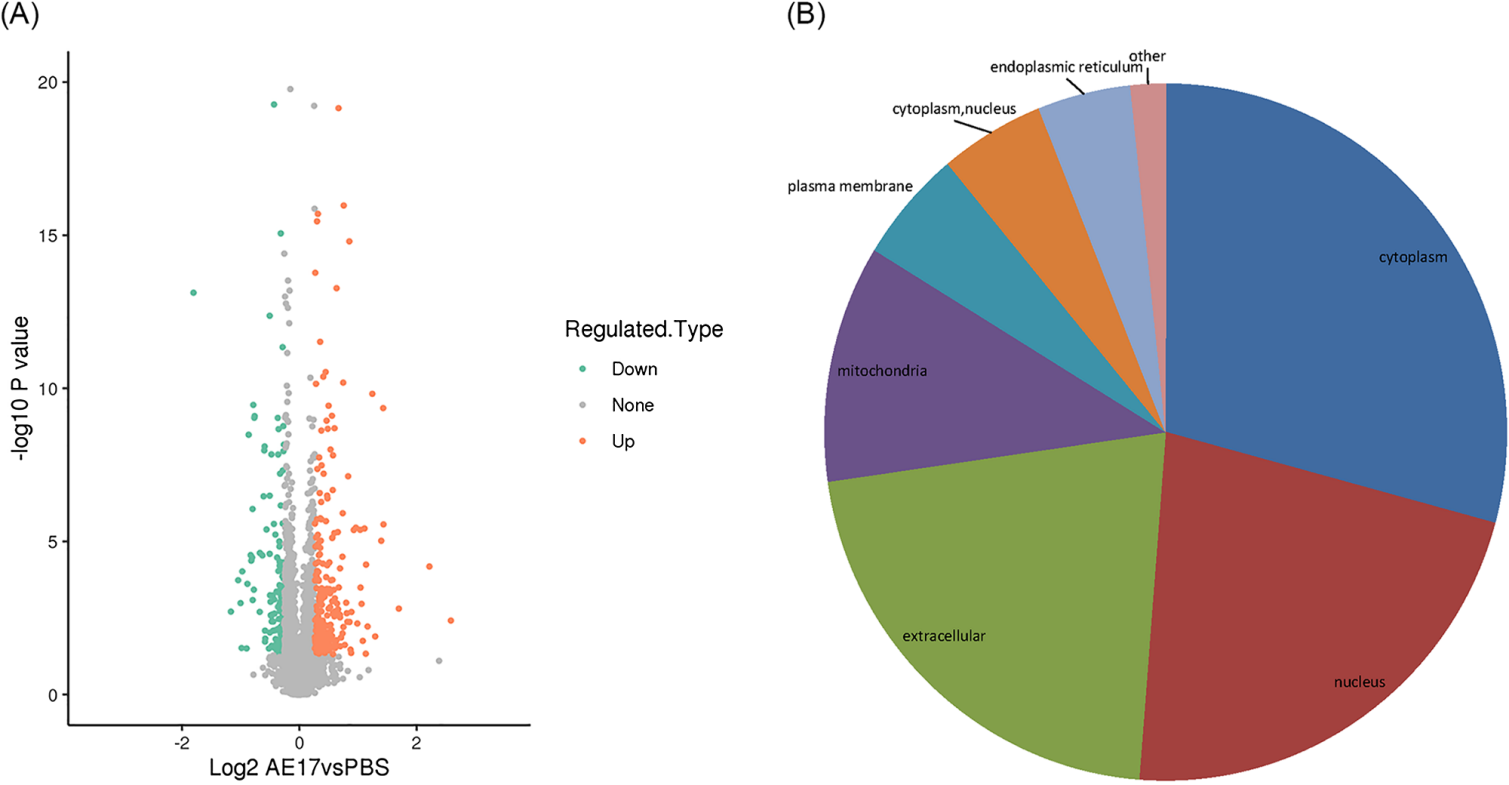
Subcellular localization and differential distribution of differentially expressed proteins. (**A**) Differential protein volcano plot after infection. (**B**) Subcellular localization of differentially expressed proteins following infection

To investigate the cause of heterophilic granulocytes infiltration, the collected tissues were subjected to TMT sequencing. Figure 2 A shows the volcano plot of differential proteins in AE17 VS PBS, which appeared differentially upregulated and downregulated, with 224 upregulated and 140 downregulated proteins. Subcellular localization showed that differential proteins occurred mainly in the cytoplasm, nucleus, extracellular sites, and mitochondria (Fig. 2B).

### Analysis of up- and downregulation of differentially expressed proteins in the GO database

**Fig. 3:**
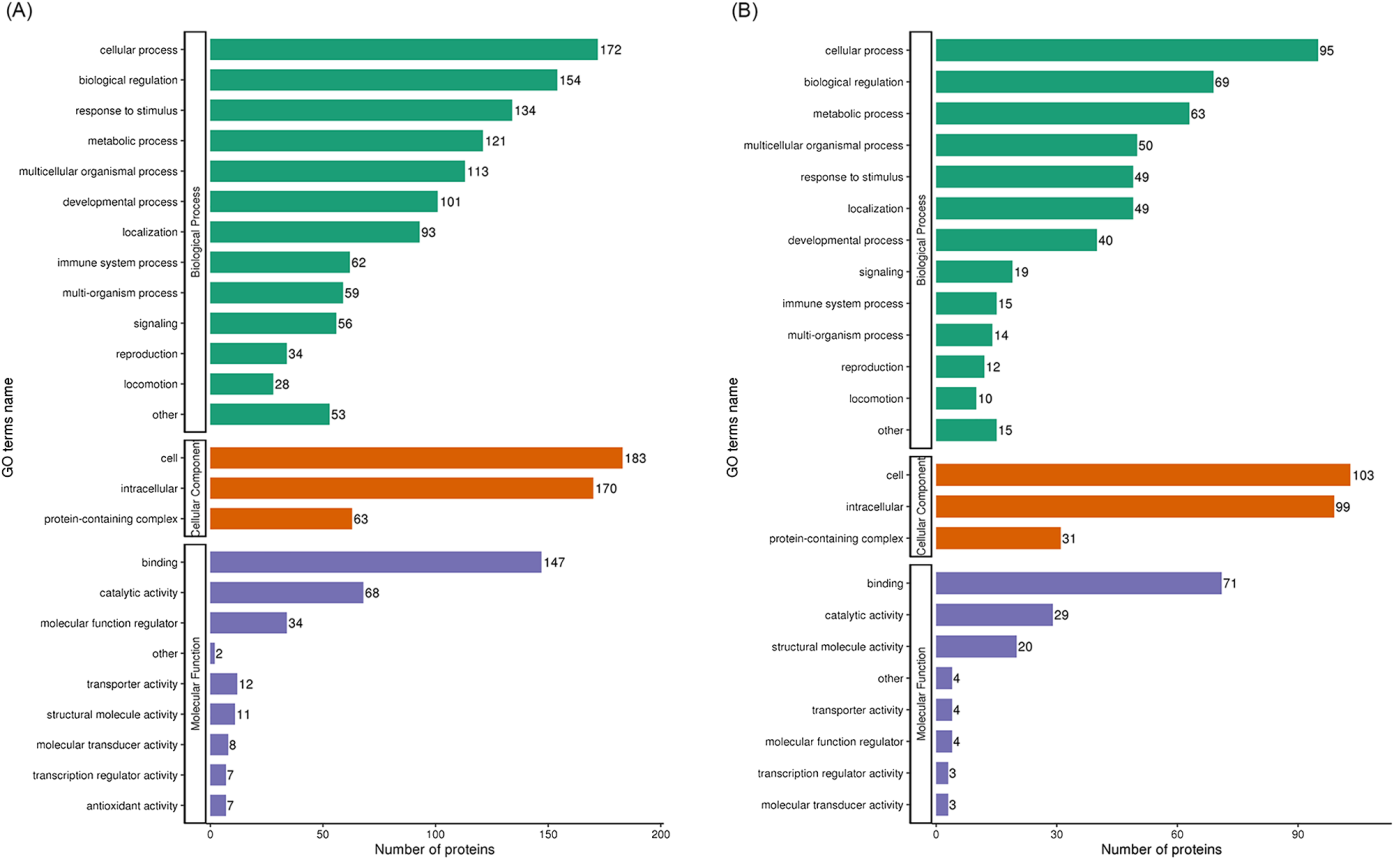
Gene ontology (GO) analysis of differentially expressed proteins for classification and annotation. (**A**) GO analysis of differentially expressed protein upregulation following infection. (**B**) GO analysis of downregulation of differentially expressed proteins following infection

We evaluated the biological functions of these differential proteins by GO analysis; GO analysis and annotation classified these differential proteins into three categories: biological processes, cellular components, and molecular functions. Regarding biological processes, there were 172 upregulated and 95 downregulated proteins in cellular processes (Fig. 3A and B); 154 upregulated and 69 downregulated proteins in biological processes (Fig. 3A and B), and 134 upregulated and 63 downregulated proteins in response to stimuli (Fig. 3A and B). The results for cellular components showed that there were 183 upregulated and 103 downregulated proteins in cells (Fig. 3A and B), 170 upregulated and 99 downregulated proteins in intracellular sites (Fig. 3A and B), and 63 upregulated and 31 downregulated proteins in protein-containing complexes (Fig. 3A and B). In terms of molecular functions, there were 147 upregulated and 71 downregulated proteins in binding (Fig. 3A and B), 68 upregulated and 29 downregulated proteins in catalytic activity (Fig. 3A and B), and 34 upregulated and 20 downregulated proteins in molecular function regulation (Fig. 3A and B). Overall, these results suggest that the cause of heterophilic granulocytes infiltration is related to multiple underlying biological processes.

### Analysis of the KEGG pathway for differential proteins causing heterophilic granulocytes infiltration

**Fig. 4:**
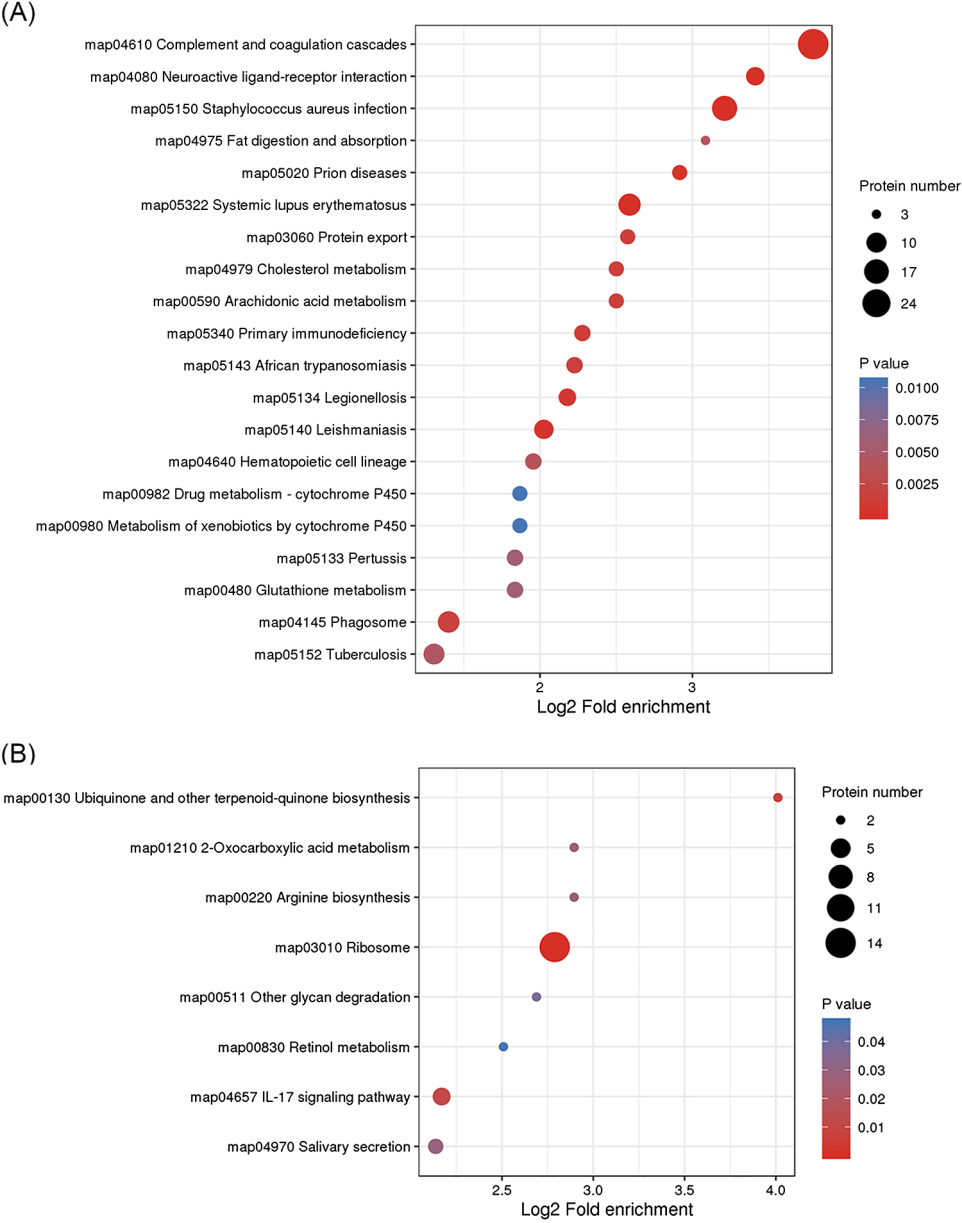
Kyoto Encyclopedia of Genes and Genomes (KEGG) analysis of differentially expressed proteins for classification and annotation. (**A**) KEGG analysis of differentially expressed protein upregulation following infection. (**B**) KEGG analysis of downregulation of differentially expressed proteins following infection

Based on the results of the KEGG pathway analysis, differential proteins in GO analysis play an important role in a number of biological pathways. The main pathways with upregulated expression were map04610 Complement and coagulation cascades, map04080 Neuroactive ligand-receptor interaction, map05150 Staphylococcal aureus infection, map04975 Fat digestion and absorption, map05020 Prion diseases, map05322 Systemic lupus erythematosus, map03060 Protein export, and map04145Phagosome (Fig. 4A). The main downregulated expression pathways were mapo00130 Ubiquinone and other terpenoid-quinone biosynthesis, map01210 2-Oxocarboxylic acid metabolism, map00220 Arginine biosynthesis, and map03010 Ribosome (Fig. 4B). Among these pathways, we focused on map04610 Complement and coagulation cascades, map03060 Protein export, map04145 Phagosome, and map03010 Ribosome. Based on our findings, the cause of eosinophil infiltration is associated with multiple differential expressions of pathways.

### Enrichment of differentially expressed proteins in complement and coagulation cascades pathways

**Fig. 5:**
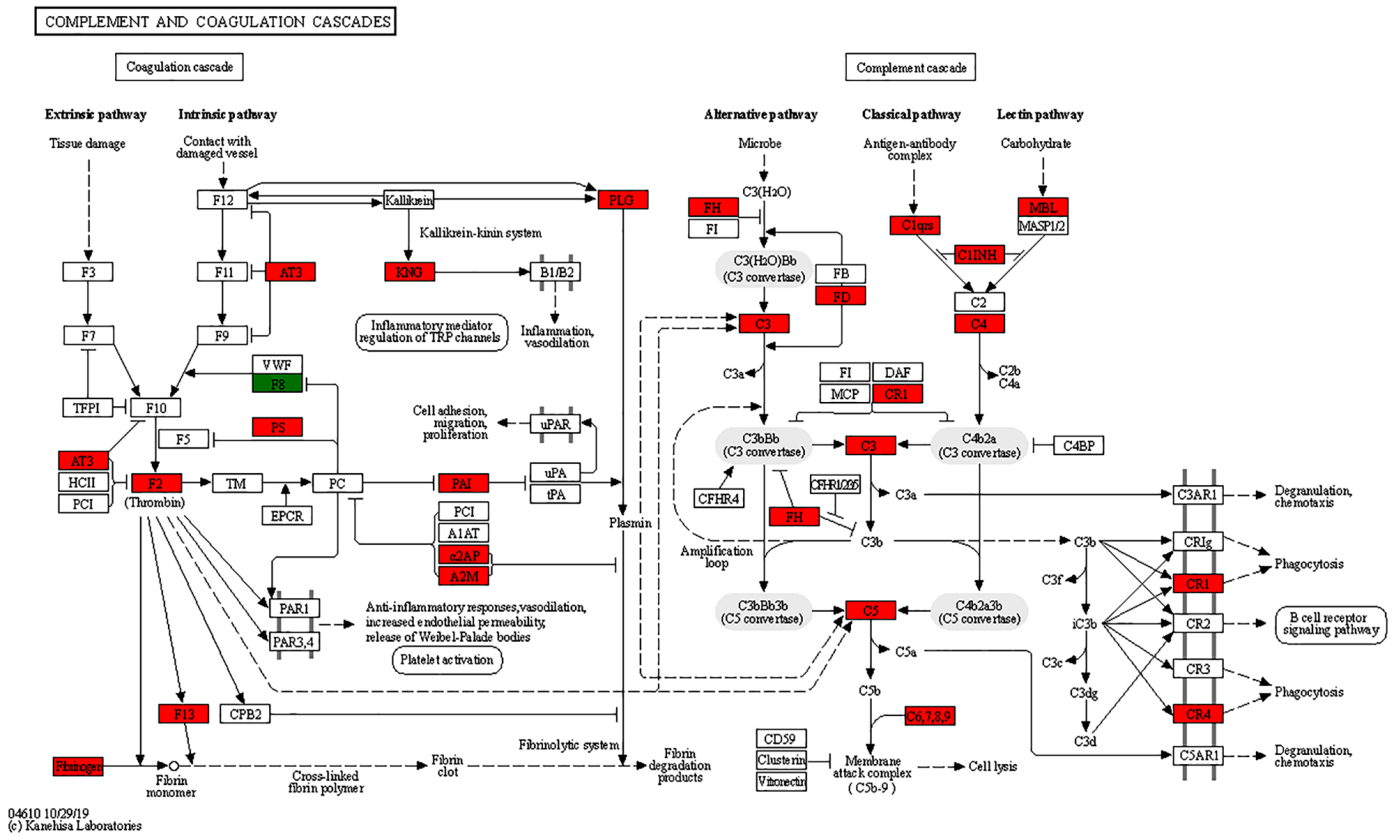
Analysis of differentially expressed proteins in Complement and coagulation cascades pathways after infection. Red is the upregulated protein; in this pathway, it is thus seen that differential proteins are upregulated, causing the inflammatory response. KEGG images have been licensed under copyright.

**Fig. 6:**
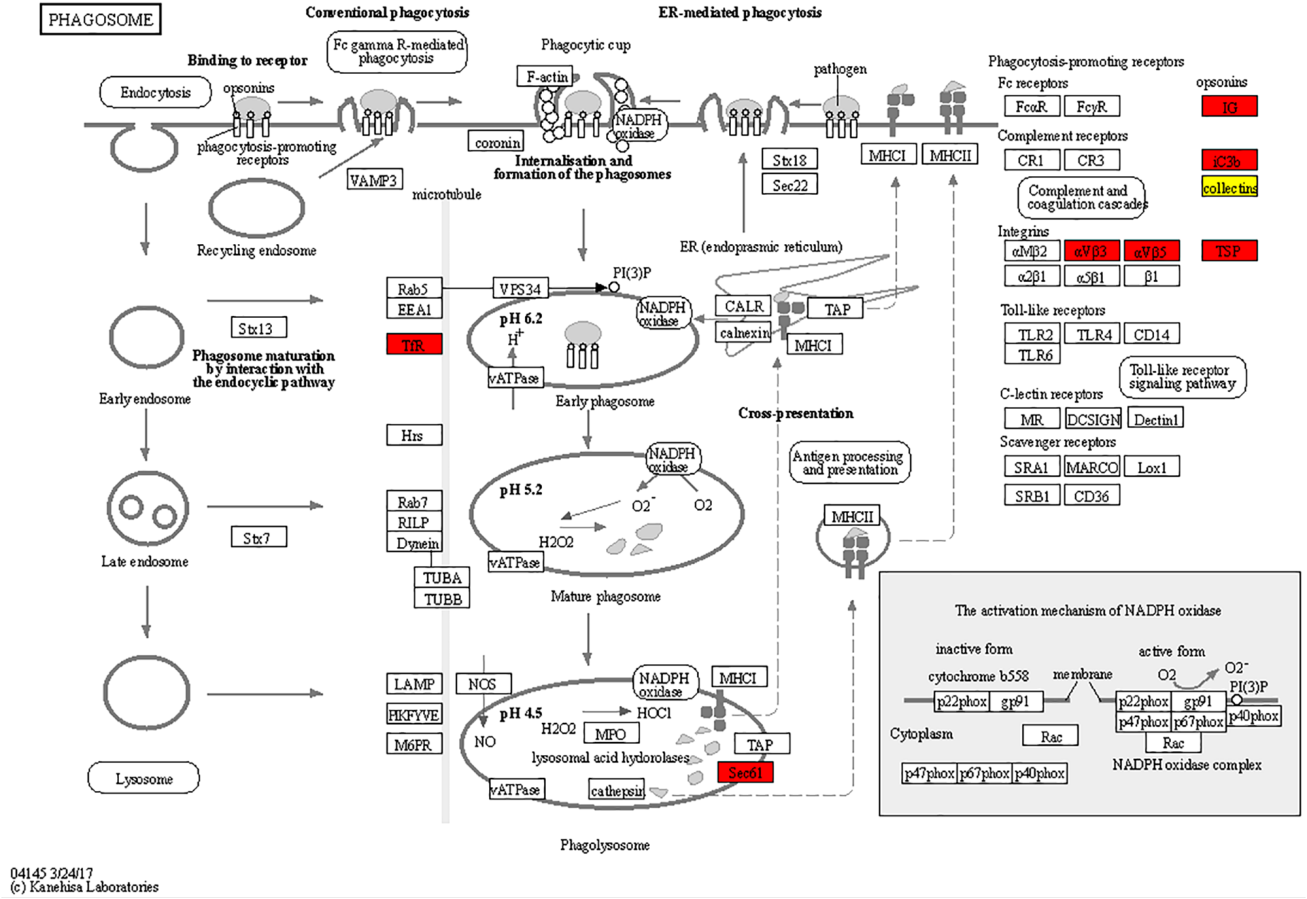
Analysis of differentially expressed proteins in Phagosome pathways after infection. Red is the upregulated protein; in this pathway, it is thus seen that differential protein upregulation enhances phagocytosis. KEGG images have been licensed under copyright.

**Fig. 7:**
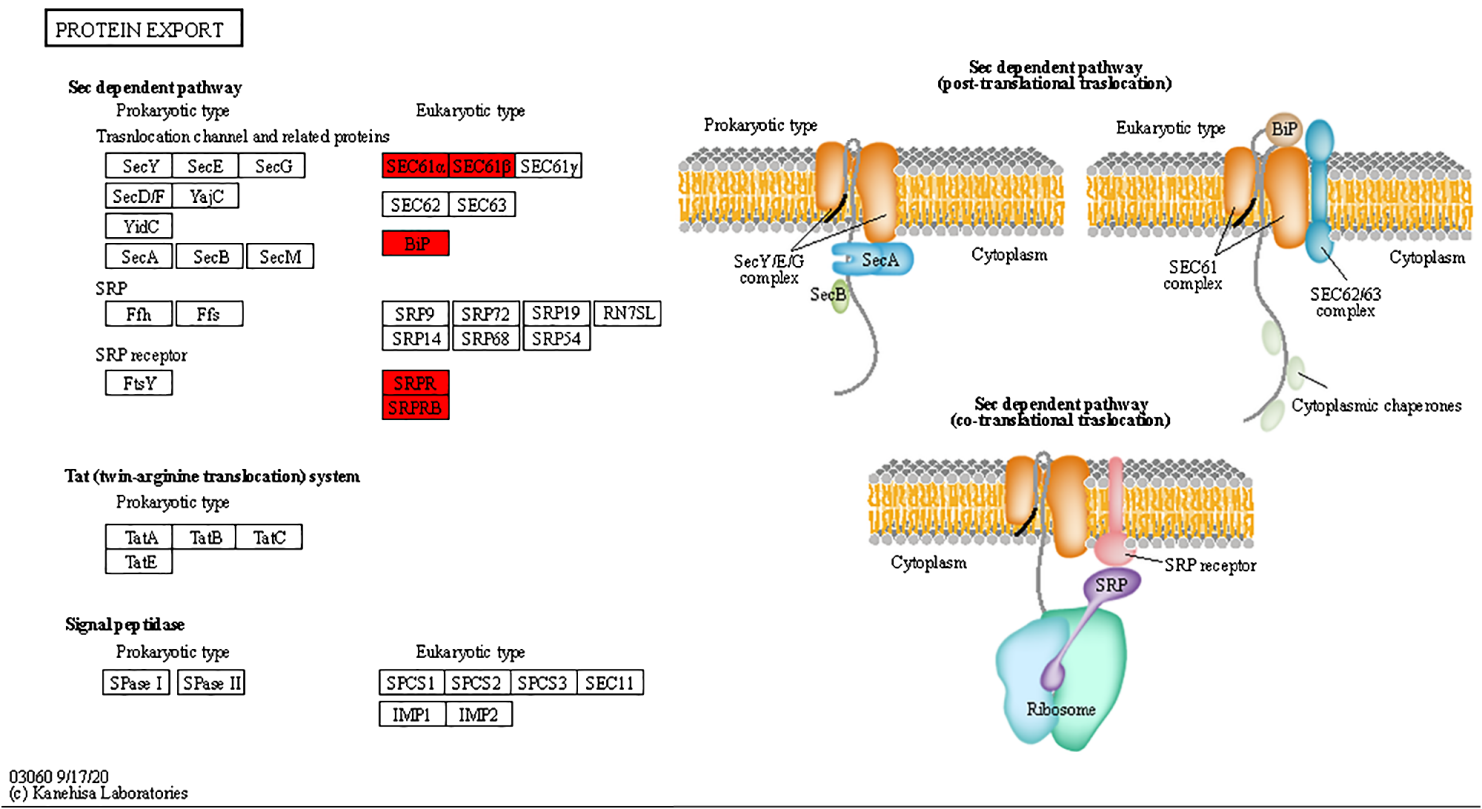
Analysis of differentially expressed proteins in Protein export pathways after infection. Red is the upregulated protein; in this pathway, visible differential protein upregulation indicates that a large amount of protein is secreted to complete the inflammation. KEGG images have been licensed under copyright.

Analysis of the results by KEGG included Complement and coagulation cascades, Phagosome, Protein export, and Ribosome pathways, suggesting that up- or downregulation of differential proteins in these pathways caused infiltration of heterophilic granulocytes. Among them, the Complement and coagulation cascades pathway showed a higher amount of differentially expressed proteins; this pathway was closely related to the inflammatory response, with a higher differential fold of differential proteins (Table 1). The expression of some key proteins in all three pathways of the complement system was upregulated (Fig. 5). When activated, the complement system can play the role of lysing bacterial viruses, promoting phagocytosis, and clearing immune complexes, in which the expression levels of C3, C4, C5, CR1, and CR4 proteins were all upregulated. In the Protein export pathway, SEC61α, SEC61β, BiP, SRPR, and SRPRB were upregulated in expression (Fig. 6). In the Ribosome pathway, 14 proteins, such as L4e, L8e, and L35e, were downregulated, and L40e protein was upregulated (Fig. 7).

**Table 1:** Differentially expressed proteins in the complement and coagulation cascades pathway

Protein accession	Gene name	Protein description	AE17/PBS ratio	Regulation type
E1BV78	FGG	Fibrinogen C-terminal domain-containing protein	2.692	Up
A0A3Q2UKP2	--	MG2 domain-containing protein (predicted)	2.450	Up
F1P4V1	FGA	Fibrinogen alpha chain	2.369	Up
A0A3Q2TZA4	--	A2M_recep domain-containing protein	2.087	Up
A0A1D5PYR9	ITGAD	VWFA domain-containing protein	1.830	Up
F1NUL9	FGB	Fibrinogen beta chain	1.805	Up
A0A3Q3AZ85	F13A1	TGc domain-containing protein	1.739	Up
E1C7C1	C8B	Complement component 8 subunit beta	1.683	Up
A0A3Q2U324	--	A2M domain-containing protein	1.663	Up
A0A1D5PWR4	C3	Complement C3 (predicted)	1.589	Up
A0A1D5PCD2	--	A2M_recep domain-containing protein	1.575	Up
E1BTH3	SERPINB2	SERPIN domain-containing protein	1.520	Up
A0A1D5PD98	C5	Complement C5 (predicted)	1.515	Up
F1NXV6	F2	Activation peptide fragment 1	1.510	Up
F1N803	C1S	Complement C1s (predicted)	1.503	Up
F1NAR5	SERPINF2	SERPIN domain-containing protein	1.477	Up
A0A1D5P9F9	C3	Complement C3 (predicted)	1.474	Up
A0A1D5PU94	C4A	C4a anaphylatoxin	1.445	Up
F1NWX6	PLG	Plasminogen	1.40	Up
A0A3Q2U7P5	CFD	Peptidase S1 domain-containing protein	1.39	Up
F1P2M6	LOC419851	Complement regulatory soluble protein (predicted)	1.388	Up
A0A3Q2TRY3	CFH	Complement factor H (Fragment) (predicted)	1.379	Up
R4GMH5	LOC428593	Plasminogen	1.374	Up
A0A1L1RNR4	KNG1	Kininogen 1 (predicted)	1.353	Up
E1C6U2	C7	Complement component 7 (predicted)	1.307	Up
F1NA58	--	Calponin-homology (CH) domain-containing protein	1.299	Up
F1NLP7	SERPINC1	Antithrombin-III	1.281	Up
Q98TA4	MBL	Mannose-binding protein	1.240	Up
F1NJU5	C8A	Complement C8 alpha chain (predicted)	1.236	Up
A0A3Q2U504	PROS1	Vitamin K-dependent protein S	1.227	Up
A0A1D5NW08	F8	Coagulation factor VIII (predicted)	0.734	Down

## Discussion

The pathogenic mechanisms of APEC are complex and diverse, causing huge economic losses worldwide [1, 11]. Studies have shown that T6SS secretes effectors that not only facilitate bacterial nutrient acquisition [12], organelle synthesis, or defense against host immunity but also assist in the precise input of virulence factors. Previous laboratory experiments confirmed that APEC infection led to pathological changes in the chicken trachea and caused the infiltration of heterophilic granulocytes [4]. In another study, infection with *Salmonella enteritidis* resulted in differential protein expression in heterophilic granulocytes and the release of granule proteins that capture and kill invading microorganisms, in vitro and in vivo [13]. Another study showed that important heterophil-mediated defense mechanisms in chickens are achieved by chicken heterophil extracellular traps (HETs) [14].

We further investigated how APEC infection caused the infiltration of heterophilic granulocytes. The pathological sections showed that heterophilic granulocytes infiltration occurred after AE17 infection of chicken trachea. Then, we sequenced the tracheal tissues of the PBS group and the AE17 experimental group by TMT proteomics and inferred the cause of heterophilic granulocytes infiltration from the sequencing results. Our results showed that differentially expressed proteins appeared after infection by AE17 to varying degrees. The GO results suggest that heterophilic granulocytes infiltration is involved in a variety of biological processes. Of interest to us, serine-type endopeptidase inhibitor activity and regulation of complement activation were particularly significant in the upregulated proteins. In the downregulated proteins, the structural constituent of ribosome and protein localization to endoplasmic reticulum processes were significant. Bioinformatics analysis showed that these differentially expressed proteins function in biological processes, cellular components, molecular functions, subcellular locations, and signaling pathways. Subcellular localization analysis showed that these differential proteins were mainly distributed in the cytoplasm, nucleus, and mitochondria. Serine protease inhibitors are widely found in animals, plants, and microorganisms. They are natural inhibitors of serine proteases and involved in many important life processes, such as coagulation, complement activation, inflammatory response, cell migration, cell matrix reconstitution, and tumor suppression; upregulation of serine protease inhibitors implies thrombosis, hemostasis, and fibrinolysis and causes fibrin production [15]. Serine protease inhibitors can also act as potent inhibitors of thrombolytic serine proteases to modulate the inflammatory response [16]. Thus, upregulation of the complement system with serine protease inhibitors is a manifestation of the involvement in the inflammatory response, indirectly causing the infiltration of heterophilic granulocytes. KEGG pathway analysis showed significant differences in up-regulated proteins of the complement and coagulation cascade, protein output and phagosome pathways, and significant differences in down-regulated proteins of the ribosomal pathway. We believe that the phenomenon causing eosinophile infiltration is related to this phenomenon, and these results are consistent with the results of GO analysis. Therefore, we believe that the phenomenon of heterophile granulocyte infiltration is related to the differential proteins in these pathways. The extraordinary ability of pathogens to use the host hemostatic system to support microbial survival and dissemination following infection by *Streptococcus pyogenes* has been previously reported [17], the coagulation cascade includes both the contact system and the tissue factor pathway, both of which lead to fibrin formation. Analysis of the Complement and coagulation cascades pathway showed high fold differences between the C3 and C4 proteins, which have a high degree of homology [18]. The C3 protein is a key complement protein in the alternative pathway [19], maintaining T-cell homeostasis in vivo and inducing the production of autocrine pro-inflammatory cytokines [20]. The complement system is a mediator of the protein hydrolysis cascade reaction and innate immunity in plasma, a non-specific defense mechanism against pathogens [21]. The main consequences of complement activation are pathogenic conditioning, inflammation, and recruitment of immunoreactive cells, as well as direct killing by pathogens. Coagulation is the conversion of another series of zymogens to serine proteases, culminating in the formation of thrombin, the enzyme responsible for the conversion of soluble fibrinogen into insoluble fibrin clots. Based on the GO analysis, upregulation of serine protease inhibitor expression. Protease-activated receptors, such as those activated by thrombin, are members of the G protein-coupled receptors and function as mediators of innate immunity [22]. The kinin-releasing enzyme-kinin system is an endogenous metabolic cascade reaction whose triggering leads to the release of vasoactive kinins (bradykinin-associated peptides) [23], which are associated with inflammatory processes. In addition, the complement body enhances the function of strong antibodies and phagocytes [24].

We therefore infer that the alternative pathway is mainly involved in the inflammatory response causing heterophil infiltration after AE17 infection; the Complement and coagulation cascades pathway also showed high levels of FGA, FGB, and FGG differential ploidy; as a fibronectin class, it can be enhanced in this process by binding to interleukins (IL), thereby increasing resistance to infection [25]. These three proteins are also present in the Neutrophil extracellular trap formation pathway, and heterophilic granulocytes are functionally similar to neutrophils, We therefore infer that the upregulated expression of these three proteins indirectly causes the infiltration of heterophilic granulocytes. Integrins as surface receptors of phagocytes can also mediate phagocytosis [26], and the upregulation of heterophilic granulocytes, such as avian phagocytes integrin protein, implies infiltration of heterophilic granulocytes. Interestingly, the differential multiplicity of iCb was higher in the Phagosome pathway, which is involved in the inflammatory response in synergy with the complement pathway. To enable B lymphocytes to change the expression of igM to igG for an optimal immune response, IGH expression is upregulated [27]. Pathogen phagocytosis is ultimately accomplished through combinatorial interactions between phagocytic receptors [28]. Through the Protein export pathway, we found that the up-regulated expression of the transport proteins SEC61α and SEC61β formed the Sect. 61 complex, which constitutes the protein conduction channel. The Sect. 61 complex plays an important role in the transmembrane transport of proteins and provides a new pathway for the transport of newly synthesized peptides to the ER lumen [29]. In addition, Sect. 61 is also a co-transporter with the lumenal protein Bip, which is an upstream protein of the antigen presentation pathway and completes the immune response with phagocytes [30]. In summary, when AE17 infects the chicken tracheal mucosa, it causes an increase in protein secretion, causing antigen presentation to stimulate the body’s defense response, mainly through the complement and coagulation system of C3 protein, involved in the alternative pathway, to participate in the inflammatory response, causing downstream integrin protein-mediated phagocytosis. Consequently, heterophilic granulocytes appear to infiltrate. Further, the ability of the complement system to respond rapidly and extensively to microbial invaders, apoptotic cells, and other threats by inducing a robust elimination response is essential for its role as a host defense and surveillance system. The identification of novel complement-mediated indications and the clinical availability of the first therapeutic complement inhibitors have also sparked new interest in the development of complement-targeted drugs, and recent developments hold great promise for both diagnosis and treatment [31]. It is therefore our hope that the present study provides a foundation for future research on the complement system.

## Conclusions

In summary, we used the TMT technique to identify the differentially expressed proteins after infection with strain AE17. These proteins were mainly enriched in Complement and coagulation cascades, Phagosome, Protein export, and Ribosome pathways, indicating that the infection was mainly resisted through the complement system after infection with strain AE17. These findings provide useful clues to elucidate the anti-infective role of the complement system after infection.

## Methods

### Ethics statement

The animal experiments were approved by the Institutional Animal Care and Use Committee of Anhui Agricultural University. The experiments conformed to the ARRIVE guidelines for laboratory animal welfare and ethics at Anhui Agricultural University.

### Animals

One-day-old Roman hens were purchased from Anhui Anqin Poultry Company Ltd (Anhui Province, China). We provided comfortable, clean cages with adequate water and feed and a temperature of 20–28 °C. Making the most of the experimental results will ensure that the poultry population can be reduced as much as possible in future studies. Animals were euthanized by intravenous injection of sodium pentobarbital (100 mg/kg) in the wings.

### Bacterial strains and culture conditions

The APEC strain (**AE17**)(serotype O2) used in this study was the same as that used in previous studies [32]; It was grown at 37 °C in Luria-Bertani (**LB**) solid medium (containing 1.5% agar) or LB broth. If necessary, we supplemented it with ampicillin (100 µg/mL) or chloramphenicol (30 µg/mL).

### Construction of a model for tracheal infection in AE17 chicks

The 7-day-old chicks were randomly grouped into three groups with six chicks each. Avian pathogenic *Escherichia coli* strain AE17 was activated in LB solid medium, and after successful activation, single colonies were incubated in LB liquid medium, followed by transfer at a ratio of 1:100 at 200 r/min and incubation at 37℃ until OD = 1.0. The bacterial solution was washed trice with PBS, and the concentration was adjusted to 1 × 10^6^ cfu/mL by taking 1 mL of the washed bacterial solution. The treated bacterial solution was injected into the chicks by tracheal injection; the injection volume and concentration were 100 µL, 1 × 10^8^ cfu/mL, 50 µL, 1 × 10^8^ cfu/mL, 100 µL, 1 × 10^6^ cfu/mL, respectively.

### Preparation of pathological sections

Chicks were dissected at 4, 8, 12, and 24 h after infection, and the trachea was sampled and fixed with 4% paraformaldehyde, followed by the preparation of paraffin sections; HE staining was performed to observe the lesions. The liver, spleen, and lungs were sampled at 24 h of infection, and pathological sections were prepared.

### TMT proteomics sequencing l stress

Tracheal mucosal epithelial cells were scraped at 12 and 24 h of infection, frozen in liquid nitrogen, and stored at -80℃. The cells were sent to a biological company (LC-bio, Hangzhou, China) for TMT quantitative proteomics analysis. Fold change > 1.2 and < 0.83, as well as a p-value of < 0.05, were used to represent up- or downregulation, respectively. These differentially expressed proteins were analyzed by bioinformatics through Gene Ontology (**GO**), Kyoto Encyclopedia of Genes and Genomes (**KEGG**), using the STRING software. The mass spectrometry proteomics data have been deposited to the ProteomeXchange Consortium via the PRIDE [33] partner repository with the dataset identifier PXD044411.

### Statistical analysis

All assays were performed at least in triplicate. All data were evaluated using Student’s t-test in SPSS ver. 17.0 (SPSS Inc., Chicago, IL, USA). A p-value less than 0.05 was considered significant.

## Data Availability

All data generated or analyzed during this study are included in this published article.
